# *NPY* Gene Methylation as a Universal, Longitudinal Plasma Marker for Evaluating the Clinical Benefit from Last-Line Treatment with Regorafenib in Metastatic Colorectal Cancer

**DOI:** 10.3390/cancers11111649

**Published:** 2019-10-25

**Authors:** Lars Henrik Jensen, René Olesen, Lone Noergaard Petersen, Anders Kindberg Boysen, Rikke Fredslund Andersen, Jan Lindebjerg, Lise Nottelmann, Caroline Emilie Brenner Thomsen, Birgitte Mayland Havelund, Anders Jakobsen, Torben Frøstrup Hansen

**Affiliations:** 1 Department of Oncology, Vejle University Hospital, 7100 Vejle, Denmark; Lise.Nottelmann@rsyd.dk (L.N.); Caroline.Emilie.Brenner.Thomsen@rsyd.dk (C.E.B.T.); Birgitte.Mayland.Havelund@rsyd.dk (B.M.H.); Anders.Jakobsen@rsyd.dk (A.J.); Torben.Hansen@rsyd.dk (T.F.H.); 2 Institute of Regional Health Research, University of Southern Denmark, 5230 Odense, Denmark; Jan.Lindebjerg@rsyd.dk; 3 Danish Colorectal Cancer Center South, Vejle University Hospital, 7100 Vejle, Denmark; Rikke.Fredslund.Andersen@rsyd.dk; 4 Department of Oncology, Aarhus University Hospital, 8000 Aarhus, Denmark; rene.olesen@rn.dk (R.O.); Anders.Kindberg.Boysen@auh.rm.dk (A.K.B.); 5 Department of Oncology 5073, Copenhagen University Hospital Rigshospitalet, 2100 Copenhagen, Denmark; Lone.Noergaard.Petersen@regionh.dk; 6 Department of Biochemistry, Vejle University Hospital, 7100 Vejle, Denmark; 7 Department of Pathology, Vejle University Hospital, 7100 Vejle, Denmark

**Keywords:** ctDNA, *NPY* methylation, biomarker, colorectal cancer, regorafenib

## Abstract

There is a need for biomarkers to improve the clinical benefit from systemic treatment of colorectal cancer. We designed a prospective, clinical study where patients receiving regorafenib as last-line treatment had sequential blood samples drawn. Effect and toxicity was monitored. The primary clinical endpoint was progression free survival (PFS). Cell-free circulating tumor (ct) DNA was measured as either the fraction with *Neuropeptide Y* (*NPY*) methylated DNA or *KRAS/NRAS/BRAF* mutated ctDNA. One hundred patients were included from three Danish centers. Among 95 patients who received regorafenib for at least two weeks, the median PFS was 2.1 months (95% confidence interval (CI) 1.8–3.3) and the median overall survival (OS) was 5.2 months (95% CI 4.3–6.5). Grade 3–4 toxicities were reported 51 times, most frequently hypertension, hand-food syndrome, and skin rash. In the biomarker population of 91 patients, 49 could be monitored using mutated DNA and 90 using methylated DNA. There was a strong correlation between mutated and methylated DNA. The median survival for patients with a level of methylated ctDNA above the median was 4.3 months compared to 7.6 months with ctDNA below the median, *p* < 0.001. The median time from increasing methylated ctDNA to disease progression was 1.64 months (range 0.46–8.38 months). In conclusion, *NPY* methylated ctDNA was a universal liquid biopsy marker in colorectal cancer patients treated with regorafenib. High baseline levels correlated with short survival and changes during treatment may predict early effect and later progression. We suggest plasma *NPY* methylation analysis as an easy and universally applicable method for longitudinal monitoring of ctDNA in metastatic colorectal cancer patients.

## 1. Introduction

Last line treatment with regorafenib for patients with metastatic colorectal cancer has proved limited survival benefit in randomized trials and a severe toxicity profile [1]. Therefore, biomarkers are essential in order to optimize the patient selection before treatment. Furthermore, biomarkers are needed for early detection of resistance, in order to help stop an inefficient treatment as early as possible.

Cell-free circulating tumor specific DNA (ctDNA) in plasma is a potential surrogate for the entire tumor genome and may be used as a “liquid biopsy” [2]. Serial blood tests with analysis of ctDNA is a promising method for both initial selection of patients to receive treatment and for monitoring treatment effect during therapy [3,4]. The fraction of the total DNA in plasma that is tumor specific can be defined as the fraction with DNA sequence mutations only present in tumor tissue. Most commonly, ctDNA is detected by next generation sequencing either directly in plasma or in tumor tissue followed by PCR analysis for quantification in plasma of specific mutations. One of the major drawbacks of this method is the pronounced heterogeneity of mutations between different colorectal tumors. 

Epigenetic changes, i.e., aberrant methylation of DNA, affect gene expression and are important in the carcinogenesis [5]. Aberrant methylation may be a more robust target for detecting and quantifying ctDNA [6,7,8], and preliminary results support this use [9]. Data from clinical settings are lacking.

The neurotransmitter Neuropeptide Y (NPY) is involved in cell motion and cell proliferation and can reduce the invasive potential of colon cancer cells in vitro [10]. The gene is frequently hypermethylated in certain carcinomas and gene promoter hypermethylation is correlated with inactivation of gene expression [11]. Roperch et al. proposed a panel of tumor-specific hypermethylated genes including *NPY* and confirmed their power to discriminate healthy individuals from patients with risk of colorectal cancer [12]. The same panel was investigated by Garrigou et al. analyzing hypermethylation in different stages of colorectal cancer to identify universal blood markers in the follow up setting [13].

The standard systemic treatments for stage IV colorectal cancer include the cytotoxic agents 5-flourouracil, irinotecan and oxaliplatin. The anti-EGFR antibodies cetuximab or panitumumab should be added in the case of *KRAS*, *NRAS,* or *BRAF* (*RAS/RAF*) wild-type tumors. Bevacizumab may be supplemented to chemotherapy, but there is no marker for optimally select patients for this agent. After exposure, intolerance or contraindications to these drugs, the patient care should be focused on supportive and palliative care or experimental treatments.

Regorafenib is an oral multikinase inhibitor which targets e.g., VEGFR1, VEGFR2, VEGFR3, TIE2, PDGF, FGFR, RET and cKIT [14]. In the pivotal phase III trial, the median survival was prolonged for 1.4 months, but with more than half of the patients experiencing grade 3+ toxicity [1].

The effect is below the bar defined by American Society of Clinical Oncology (ASCO) for clinically meaningful outcomes [15] and turns into an unfavorable score in European Society for Medical Oncology’s (ESMO’s) approach to stratify drugs based on the magnitude of clinical benefit [16]. In Denmark, the drug has not been approved by the authorities for standard use.

Based on a proven anti-cancer effect, we wanted to investigate the outcomes from regorafenib treatment in a Danish colorectal cancer patient cohort. Because of the expected limited efficacy, we also wanted to evaluate ctDNA as a marker which may help to identify patients with expected no or minimal effect of regorafinib. We hypothesized that tumor specific methylation of *NPY* in plasma DNA correlated with ctDNA measured with DNA nucleotide mutation. Furthermore, we hypothesized that *NPY* methylation changes during regorafenib treatment reflected the clinical course and could predict progression earlier than imaging. 

## 2. Results

### 2.1. Patient Characteristics

**Figure 1:**
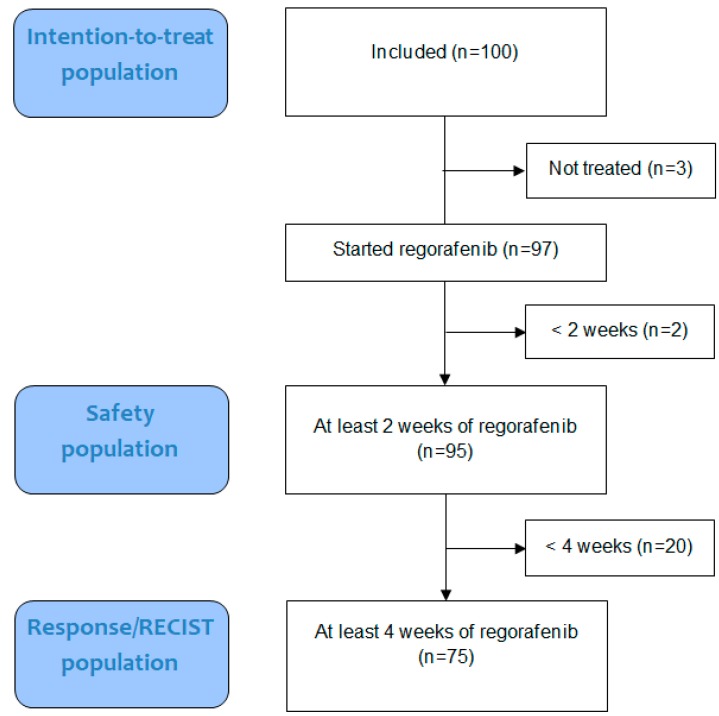
Patient Flow with an illustration of the intention-to-treat, safety, and Response evaluation criteria in solid tumors (RECIST) population.

From October 2013 to May 2016, 100 patients were included. The patient flow is shown in Figure 1. Most patients were in performance status 1 (*n* = 54) and 43 in performance status 0. Performance status was not specified as 0 or 1 in three cases. Patient characteristics are shown in Table 1.

### 2.2. Treatment

Three patients were included but never received treatment. The 97 patients starting regorafenib received it for a median of 63 days (range 3 to 493 days). 

### 2.3. Toxicity

Ninety-five patients were treated with regorafenib for at least two weeks and were considered evaluable for toxicity. Grade 3–4 toxicities were reported 51 times, most frequently hypertension, hand-food syndrome, and skin rash. Details of toxicity are given in Table 2.

### 2.4. Efficacy of Regorafenib

Patients were evaluable for response if they received regorafenib for at least four weeks and had measurable disease on the baseline scan. Seventy-five patients fulfilled these criteria, 60% had stable disease and 40% had progression as best response. There were no complete or partial responses. 

**Figure 2:**
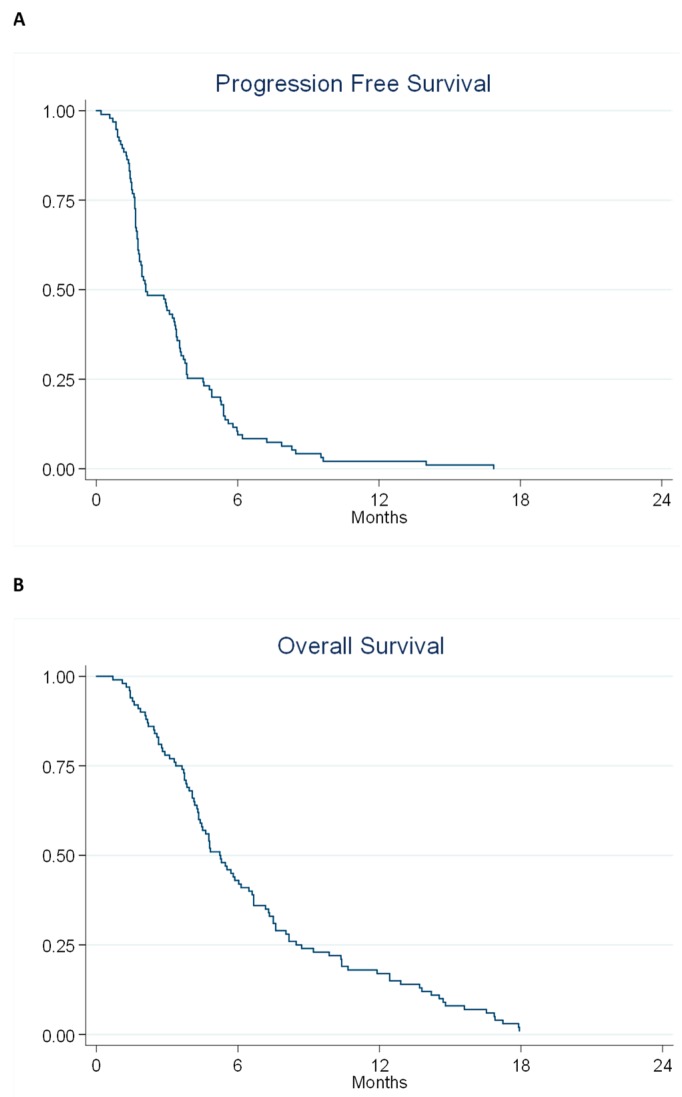
Kaplan–Meier Plots describing progression free survival (**A**) and overall survival (**B**) for the entire cohort.

All patients were evaluable for PFS and OS and all but two patients died during followed up. The median PFS was 2.1 months (95% confidence interval (CI) 1.8–3.3) and the median OS was 5.2 months (95% CI 4.3–6.5). The PFS and OS curves are shown in Figure 2.

Forty-six patients were alive without progression at two months. Thus, the fraction of PFS at two months was 46% (95% CI 36.1–55.9%) of the intention-to-treat population. Efficacy data is summarized in Table 3.

### 2.5. Tumor Specific DNA

Plasma samples were available from 91 patients (anytime) and 82 at baseline. In 49 of 61 patients with a known tumor *RAS/RAF* mutation the same mutation was detected in at least one plasma sample. In 90 of 91 patients, methylated *NPY* was detected at least in one sample. The case where *NPY* methylation failed was a patient with a single blood sample who stopped regorafenib after three days, i.e., not eligible for toxicity or response evaluation.

**Figure 3:**
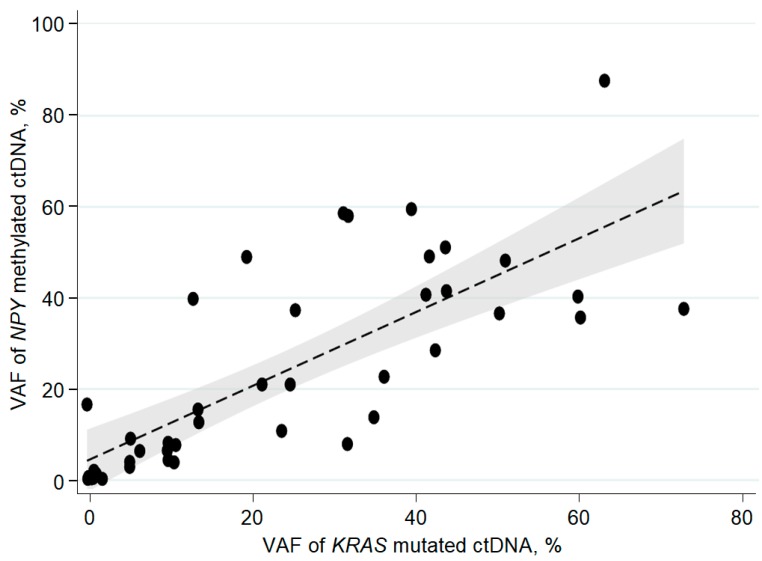
Correlation between baseline cell-free circulating tumor specific DNA (ctDNA) in the *RAS* mutation cohort measured by mutation or methylation, Spearman’s rho = 0.82, *p* < 0.001, *n* = 42. VAF = Variant allele frequency, dashed line is the linear regression line with 95% confidence interval in grey.

There was a strong correlation between paired observations of fraction of *RAS/RAF* mutation and fraction of *NPY* methylated ctDNA; Spearman’s rho was 0.82 (*p* < 0.001) for paired observations at baseline (Figure 3). Correspondingly, Spearman’s rho was 0.83 and 0.87 for the two next sampling time points during therapy (*p* < 0.001).

The fraction of *RAS/RAF* mutated ctDNA and the fraction of *NPY* methylated ctDNA were independent from sex, age, and PS at baseline. Table 4 depicts the biomarker population and correlations with basic characteristics.

There was a negative correlation between the level of baseline mutated ctDNA and the primary endpoint PFS at two months, *p* = 0.004 (*n* = 46), but not for the fraction of methylated ctDNA, *p* = 0.26 (*n* = 82).

**Figure 4:**
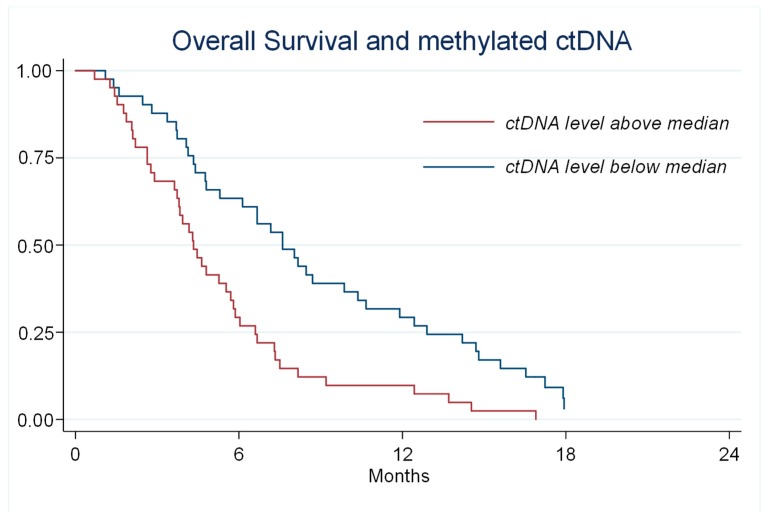
Kaplan–Meier Plots describing overall survival for patients with a level of methylated circulating tumor (ct) DNA above or below the median. Median survival was 7.6 vs. 4.3 months, *p* < 0.001.

The fraction of ctDNA at baseline correlated with OS both for mutated ctDNA, *p* < 0.001 (*n* = 46) and for methylated ctDNA. The median OS for patients with a level of methylated ctDNA above the median was 4.3 months compared to 7.6 months with ctDNA below the median, *p* < 0.001 (Figure 4). In a multivariate model including age, sex, location of primary tumor, performance status, and level of methylated ctDNA, the latter was the only significant predictor of survival (*p* = 0.001). 

### 2.6. Dynamics of ctDNA

The initial effect of regorafenib on ctDNA was evaluated by comparing 74 patients who had a baseline sample and a follow-up sample. The fraction of *NPY* methylated DNA fell initially in 68 of 74 patients (92%).

**Figure 5:**
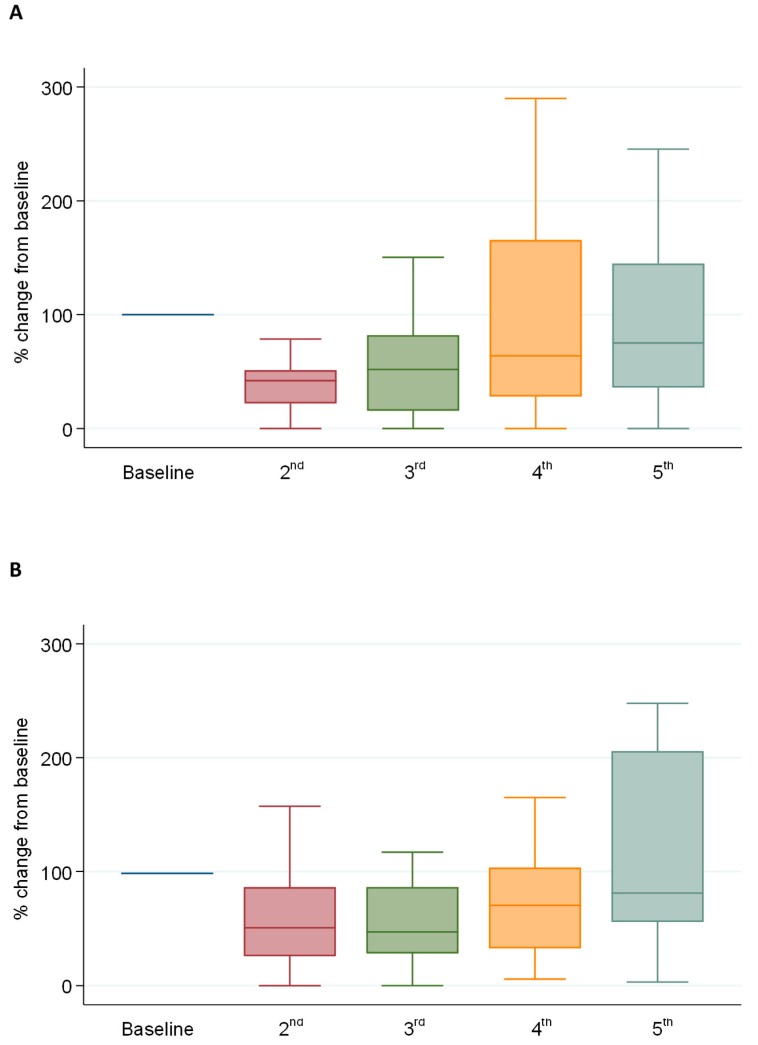
(**A**) methylated circulating tumor (ct) ctDNA and (**B**) mutated ctDNA. Baseline and five serial plasma samples were available from 40 and 23 patients, respectively. The boxplots of medians show the longitudinal changes of normalized values of the fraction of ctDNA.

The longitudinal changes during treatment were described by normalizing the fraction of methylated ctDNA to 100 at baseline for patients with at least five serial plasma samples (*n* = 38). The relative change during treatment is shown in Figure 5A. There is an immediate decline initially followed by an increase. The mean normalized fraction of methylated ctDNA was 100%, 48%, 71%, 185%, and 220% for the first five samples, respectively, with a significant decline in Sample 2 and 3 compared to the baseline (*p* < 0.001 and *p* = 0.04). For comparison, the same is shown in Figure 5B for *RAS/RAF* mutated ctDNA (*n* = 23) with a similar pattern. The means were 100%, 60%, 69%, 128%, and 198% for Sample 1–5. The decrease was significant for Sample 2 and 3 compared to the baseline (*p* < 0.001 and *p* = 0.04).

Collection of longitudinal samples made it possible to evaluate the time span for individual patients from the increase in *NPY* methylated ctDNA during treatment to time of radiologic progression. Fifty-three patients evaluable according to response evaluation criteria in solid tumors (RECIST) received more than one full cycle of regorafenib and had at least three plasma samples analyzed. Patients were followed in the protocol until progression, and 48 of these 53 patients (91%) showed an increase in the fraction of *NPY* ctDNA before progression according to RECIST. The median time from increasing ctDNA to RECIST progression was 1.64 months (range 0.46–8.38 months).

## 3. Discussion

In this trial of last-line regorafenib in metastatic colorectal cancer patients, we showed that monitoring of the disease during treatment was possible using ctDNA. Most commonly, the detection of tumor specific mutations in the DNA is used to define tumor specificity. Early reports have pointed towards *NPY* methylation as a marker of tumor specificity. We show for the first time in last-line treatment with regorafenib that measuring *NPY* methylated ctDNA is superior to measuring *RAS/RAF* mutated ctDNA, as it can be measured in almost all patients irrespective of mutational status. Furthermore, the dynamics of *NPY* methylated ctDNA during treatment reflect the dynamics of *RAS/RAF* ctDNA. A smaller study has reached the same conclusion in a mixed population of patients with metastatic colorectal cancer [9]. Future studies should focus on evaluating *NPY* ctDNA and treatment effect in larger, prospective trials. 

The main clinical endpoint in the present trial was the fraction of PFS at two months (46%). The median PFS was 2.1 months and median OS was 5.2 months. In the CORRECT [1] and the CONCUR [17] trials, the median PFS was 1.9 and 3.2 and median OS 6.4 and 8.8 months, respectively. In conclusion, our data does not support a clinically relevant benefit or value [15,16] of regorafenib in unselected Danish colorectal cancer patients after standard systemic treatment for metastatic disease.

Given the low clinical effect in an unselected cohort, the question is whether defined subgroups may benefit. We used ctDNA to group patients and monitor the disease during treatment. By dividing the patients according to the median level of *NPY* methylated ctDNA, it was possible to identify a subgroup of patients with a low median OS of 4.3 months (Figure 4). These patients would probably benefit more from palliative care than specific antineoplastic treatment with the risk of toxicity [18].

In the biomarker study associated with the CORRECT trial, the relative effect, but not the absolute effect, of regorafenib was maintained across subgroups of patients divided by high or low concentration of total circulating DNA [19]. Furthermore, ctDNA, defined as level of *KRAS* mutation, had a prognostic effect. Our data supports their findings in the minority with detectable *RAS/RAF* mutations. In addition, our data adds further information that *NPY* methylated ctDNA is detectable in practically every colorectal cancer patient, correlates with *RAS/RAF* mutated ctDNA, and has the same prognostic information. This calls for trials evaluating deselection of patients with high ctDNA for last-line treatment.

Another interesting observation was the initial decrease in ctDNA in almost all patients. It can be speculated that regorafenib has a specific antineoplastic effect in the vast majority of colorectal cancer patients, but redundancy in cancer [20] leads to early resistance. If this is true, then personalized, targeted medicine, in the sense that pretreatment marker analysis can predict clinical effect [21], is destined to fail. What decides clinical benefit may instead be mechanisms of resistance [20]. We suggest performing studies in which predictions of clinical benefits of a drug is based not on a snapshot of molecular biology but on biological systems incorporating the temporal effects. These systems are under development using 3D tumor cell cultures combined with sensitivity analysis [22].

Traditionally, the duration of benefit from a drug is reflected by PFS. We showed that progression according to RECIST was preceded by an increase in ctDNA with a median of 1.6 months. Thus, ineffective treatment may be stopped earlier if based on increasing *NPY* ctDNA. We suggest that future trials should test early termination based on increasing ctDNA compared to RECIST progression with the perspective of sparing the patients from ineffective treatments and unnecessary toxicity.

The limitations of our study are primarily caused by the relatively small patient number. It is not possible to give narrow estimates of efficacy of regorafenib in just 100 patients, and subgrouping based on markers is even more uncertain. Furthermore, there is no untreated control group. Subsequently, our conclusions and suggestions based on the results are made with caution.

## 4. Materials and Methods 

### 4.1. Patients

Patients with histologically confirmed metastatic adenocarcinoma of the colon or rectum were included from three Danish cancer centres. Refractory disease was required and it was defined as progressive disease within six months of the last administration of each drug or regimen including fluoropyrimidine, oxaliplatin, irinotecan, and bevacizumab. In cases with *RAS/RAF* wild-type tumor, progression of treatment with cetuximab or panitumumab was also mandatory. If there were contraindications to any of the drugs, resistance to that specific drug was not required. Contraindications could be grade 2 or higher neurotoxicity after previous adjuvant oxaliplatin, cardiotoxicity to fluoropyrimidine, or hypersensitivity reactions despite pretreatment. Imaging performed within one month of inclusion should be evaluable, i.e., not necessarily with measurable disease, according to RECIST version 1.1 [23]. Other inclusion criteria were performance status 0–1, adequate organ function based on laboratory tests, age of at least 18 years, and informed consent. Patients were excluded in the case of uncontrolled hypertension or proteinuria, symptomatic medical conditions requiring prompt interventions, or pregnancy including chance of pregnancy and breast feeding.

### 4.2. Ethics and Approvals

The trial was approved by the Regional Ethics Committee on Health Research, Region of Southern Denmark No. S-20130057, 2013 and reported to the Danish Data Protection Agency.

### 4.3. Trial Design and Treatment

In this single-arm, prospective phase II biomarker trial, eligible patients received an initial dose of 120 or 160 mg regorafenib once daily according to institutional practice. The starting dose of 120 mg was allowed to escalate to 160 mg if tolerable. One cycle was defined as 21 days with regorafenib followed by seven days without regorafenib. Treatment continued until progression or unacceptable toxicity including patient request for stopping. Procedures for dose modifications and pausing of regorafenib were specified in the protocol according to observed toxicity.

Reporting of the study followed the Reporting Recommendations for Tumor Marker Prognostic Studies (REMARK) [24] whenever relevant.

### 4.4. Evaluations

Hematology, biochemistry, and organ function tests (Stivarga, Summary of Product Characteristics, European Medicines Agency, 2013) were performed every second week for two months and then monthly if stable. Imaging of the chest and abdomen and disease evaluation according to RECIST 1.1 was performed every second cycle of treatment.

### 4.5. Blood Samples and Tumor Tissue 

Before treatment, after two weeks of treatment, and then before every new cycle, 9 mL of blood was drawn in EDTA tubes for marker analyses. After treatment ended and until progression, blood was drawn before every evaluation scan. The samples were centrifuged at 2000 *g* for 10 minutes and stored at −80 degrees Celsius within four hours. Beside plasma samples, serum, buffy coat, and tumor biopsies were collected and stored in a biobank. 

#### 4.5.1. DNA Purification

DNA was purified from 4 mL plasma after thawing and centrifugation for 10 min at 10,000 *g* using QIAsymphony Circulating DNA kit (Qiagen, Venlo, The Netherlands). An internal control, 20 µL *CPP1*, was added [25] and the DNA was eluted in 60 µL before the addition of 340 µL water. The QIAsymphony robot (Qiagen) was used for purification. 

#### 4.5.2. Quantification of Total Plasma DNA

Quantitative PCR analysis of *B2M* and *CPP1* was performed on a Quantstudio 12k Flex real-time PCR system (Life Technologies, Waltham, MA, USA) according to [25]. DNA from lymphocytes and water were included as external controls in the qPCR.

#### 4.5.3. Concentration

In samples where both *RAS/RAF* mutation and methylation analysis were performed, DNA was concentrated to 25 µL using Amicon Ultra-0.5 Centrifugal Filter Unit (Merck Millipore, Burlington, MA, USA), and 12 µL was used for mutation analysis and 12 µL for bisulfite conversion. If only methylation analysis was performed, half of the DNA was concentrated to 20 µL. In samples with high total DNA, the amount of DNA for conversion was reduced accordingly.

#### 4.5.4. RAS/RAF Mutation Analysis

The specific *RAS/RAF* mutation known from the tumor tissue analysis was analyzed in duplicates with five µL samples in 20 µL reactions using digital droplet (dd) PCR supermix for probes (no dUTP) and Bio-Rad PrimePCR assays. Controls included gBlock mixed with wild-type donor DNA as positive control and wild-type donor DNA and water as negative controls for each mutation assay. Droplets were generated with QX200 Automated Droplet Generator (Bio-Rad, Hercules, CA, USA) and PCR run on a Veriti thermal cycler (Applied Biosystems, Waltham, MA, USA). The QX100 Droplet Digital Reader (Bio-Rad) was used for reading of the samples.

#### 4.5.5. Methylation Analysis

DNA underwent bisulphite conversion in a 50 µL reaction using EZ DNA Methylation Lightning Kit (Zymo Research, Irvine, CA, USA) before eluting to 12 µL. NPY/Albumin analysis was done according to Garrigou [13] with ddPCR as described above. Data analysis was completed with QuantaSoft ver. 1.7.4 (Bio-Rad).

All analyses were performed by personal blinded to the study end points.

### 4.6. Statistics

The clinical primary endpoint was the fraction of progression free survival (PFS) at two months defined as patients alive without progression at the radiological evaluation after two cycles divided with the number of all patients who entered the trial on an intention-to-treat principle. It was anticipated that 50% of the patients would progress at two months [1]. We considered it of clinical interest to detect a 15% point change in PFS at two months. With a power of 90% and a 5% risk of a type-one error, 56 of 97 included patients should be without progression two months after inclusion to indicate an effect of 15% point [26]. Hence, we aimed at including 100 patients to allow for a clinical test and a biomarker evaluation.

Two central experimental plasma biomarkers were evaluated in this study. The first was ctDNA measured as the fraction of mutated ctDNA with specific *RAS/RAF* sequence mutation relative to total DNA in plasma. The second was ctDNA measured as the fraction of ctDNA with methylated *NPY* gene relative to total DNA in plasma. Spearman’s rank-order correlation described the correlation between these two biomarkers.

Baseline characteristics such as age, sex, performance status, histopathology, date of diagnosis, and type of previous treatments were described using descriptive statistics. Toxicity was evaluated at baseline and at every cycle using common terminology criteria for adverse events version 4 (National Cancer Institute 2009), and the worst grade was noted. Treatment was described by dates of first and last administration, dose, and dose modifications. The effect of regorafenib was measured according to RECIST 1.1 [23] with respect to response and progression. PFS was defined as the time from inclusion in the trial to the first date of progression or death from any cause. Overall survival (OS) was defined as the time from inclusion to death independent from cause. The Kaplan–Meier method was used for PFS and OS in this setting and comparisons were done with the log rank test. Patients who received at least two weeks of regorafenib were evaluable for toxicity and those receiving at least one cycle were evaluable for response. All patients were evaluable for PFS and OS according to the intention-to-treat principle. Level of significance was 5% and calculations were done using the statistical software STATA version 14.0 (StataCorp, College Station, TX, USA).

## 5. Conclusions

In conclusion, *NPY* methylation and *RAS/RAF* mutation analysis to determine ctDNA are interchangeable, but *NPY* is superior as it can quantify ctDNA in nearly all the patients. High baseline levels correlate with short survival, and monitoring plasma tumor DNA using *NPY* methylation during treatment may predict early effect and later progression. We suggest plasma *NPY* methylation analysis as an easy and universally applicable method for longitudinal monitoring of ctDNA in metastatic colorectal cancer patients.

## Figures and Tables

**Table 1:** Patient characteristics for the intention-to-treat population of 100 patients. NR = not reported.

Characteristic	Categories	Data
Age	Median (min–max), years	65 (28–77)
Time since first chemotherapy for metastatic colorectal cancer	Median (min–max), years	1.8 (0.4–10.9)
		*n*
Sex	Female	47
	Male	53
Localization of primary tumor	Right colon	30
	Left colon	31
	Rectum	35
	NR	4
No of metastatic sites	1–2	54
	2+	46
Performance status	0	43
	1	54
	NR	3
Treatment, adjuvant	Fluoropyrimidine monotherapy	36
	Fluoropyrimidine and oxaliplatin	30
Treatment, metastatic	Fluoropyrimidine	96
	Oxaliplatin	87
	Irinotecan	98
	Bevacizumab	90
	EGFR inhibitor	33
	Other	5
	NR	2
Mutation in tumor tissue	*KRAS*	51
	*NRAS*	4
	*BRAF*	6
	Wild-type or NR	39

**Table 2:** Toxicity analysis of 95 patients evaluable for toxicity. The most common toxicities are listed according to frequency of minor toxicity, grade 1–2, and major toxicity, grade 3–4.

Adverse Event	Grade 1–2 *N* (%)	Grade 3–4 *N* (%)
Hypertension	31 (33%)	8 (8%)
Hand-foot-skin reaction	48 (51%)	6 (6%)
Rash	20 (21%)	6 (6%)
Fatigue	77 (81%)	4 (4%)
Diarrhea	35 (37%)	3 (3%)
Anorexia	60 (63%)	2 (2%)
Oral mucositis	43 (45%)	2 (2%)
Nausea	35 (37%)	1 (1%)
Voice changes	66 (69%)	0 (0%)
Conjunctivitis	19 (20%)	0 (0%)
Bleeding	16 (17%)	0 (0%)
Other toxicites	65 (68%)	19 (20%)

**Table 3:** Clinical Outcomes. Response rates are given for 75 patients evaluable for response. PFS = progression free survival at 2 months is the fraction of all patients alive without progression at the radiological evaluation after two cycles. Median PFS and OS = overall survival is given for the intention-to-treat population and reported using the Kaplan–Meier method.

Endpoint	Categories	*N*	Data	95% CI
Response rate	Stable disease	45	60%	48–71%
	Progressive disease	30	40%	29–52%
PFS	At 2 months	46	46%	36–56%
	Median	100	2.1 months	1.8–3.4
OS	Median	100	5.2 months	4.3–6.5

**Table 4:** Patient characteristics of the biomarker population. Correlation between patient characteristics and the mean level of circulating tumor DNA (ctDNA) at baseline determined either by *RAS/RAF* mutation (Mut ctDNA) or *NPY* methylation (Meth ctDNA) in the biomarker population, *n* = number, CI = confidence interval. In 82 patients a baseline plasma sample was available and the correlations are based on these patients.

Characteristic	Categories	Clinical Trial, *n*	Mut ctDNA			Meth ctDNA		
			*n*	Mean % (95%CI)	*p*-Value	*n*	Mean % (95%CI)	*p*-Value
Age	Median (min–max), years 65 (28–77)	100	46	25 (19–32)	0.19	82	25 (20–30)	0.24
Sex	Female	47	21	25 (14–36)		40	21 (14–27)	
	Male	53	25	25 (18–33)	0.77	41	30 (22–37)	0.11
Localization of primary tumor	Right colon	30	18	26 (16–37)		24	30 (19–44)	
	Left colon	31	14	27 (12–41)		24	21 (10–31)	
	Rectum	35	13	23 (12–35)	0.98	31	23 (17–30)	0.32
Performance status	0	43	22	24 (15–33)		34	21 (15–30)	
	1	54	24	27 (17–36)	0.96	46	27 (19–34)	0.58
